# Past mercury exposure and current symptoms of nervous system dysfunction in adults of a First Nation community (Canada)

**DOI:** 10.1186/s12940-022-00838-y

**Published:** 2022-03-16

**Authors:** Aline Philibert, Myriam Fillion, Judy Da Silva, Tanya Suvendrini Lena, Donna Mergler

**Affiliations:** 1 Université du Québec à Montréal, Centre de recherche interdisciplinaire sur le bien-être, la santé, la société et l’environnement (Cinbiose), CP 8888, Succ. Centreville, Montréal, Québec H3C 3P8 Canada; 2 Département Science et Technologie, Université TÉLUQ, 5800 Saint Denis St, Montréal, Québec H2S 3L4 Canada; 3 Grassy Narrows First Nation, General Delivery, P0X 1B0, Grassy Narrows, Ontario Canada; 4 Women’s College Hospital, 76 Grenville St, Toronto, Ontario M5S 1B2 Canada

**Keywords:** Mercury, Past exposure, First nation, Nervous system dysfunction, Symptom clusters, Clustering, Structural equation modelling, Mixed effects model

## Abstract

**Background:**

The watershed in Asubpeeschoseewagong Netum Anishinabek (Grassy Narrows First Nation) territory has been contaminated by mercury (Hg) since 1962, resulting in very high Hg concentrations in fish, central to the community’s culture, traditions, economy and diet. Biomarkers of Hg exposure (umbilical cord blood and hair/blood samples), monitored between 1970 and 1997, decreased over time. A recent Grassy Narrows Community Health Assessment (GN-CHA) survey included current symptoms of nervous system dysfunction. The present study aimed to cluster self-reported symptoms and examine their associations with past Hg exposure.

**Methods:**

The GN-CHA included 391 adults. Symptom clustering used a two-step segmentation approach. Umbilical cord Hg and/or yearly measurements of equivalent hair Hg were available for 242 participants. Structural Equation Models (SEM) displayed the associations between Hg exposure and clusters, with Hg exposure modelled as a latent variable or in separate variables (prenatal, childhood and having had hair Hg ≥ 5 μg/g at least once over the sampling period). Longitudinal Mixed Effects Models (LMEM) served to examine past hair Hg with respect to clusters.

**Results:**

A total of 37 symptoms bonded into 6 clusters, representing Extrapyramidal impairment, Sensory impairment, Cranial nerve disturbances, Gross motor impairment, Neuro-cognitive deficits and Affect/Mood disorders. Median Hg concentrations were 5 μg/L (1–78.5) and 1.1 μg/g (0.2–16) for umbilical cord and childhood hair, respectively. More than one-third (36.6%) had hair Hg ≥ 5 μg/g at least once. In SEM, latent Hg was directly associated with Extrapyramidal and Sensory impairment, Cranial nerve disturbances and Affect/Mood disorders. Direct associations were observed for prenatal exposure with Affect/Mood disorders, for childhood exposure with Extrapyramidal impairment and Cranial nerve disturbances, and for hair Hg ≥ 5 μg/g with Extrapyramidal and Sensory impairment. For all clusters, a further association between past Hg exposure and symptom clusters was mediated by diagnosed nervous system disorders. LMEM showed higher past hair Hg among those with higher scores for all clusters, except Affect/Mood disorders.

**Conclusion:**

Our findings provide evidence that in this First Nation community, past Hg exposure from fish consumption was associated with later-life clusters of coexisting symptoms of nervous system dysfunction.

**Supplementary Information:**

The online version contains supplementary material available at 10.1186/s12940-022-00838-y.

## Background

In the 1960’s, a chloralkali plant discharged approximately 10,000 kg of mercury (Hg) into the Wabigoon-English River system in Northwestern Ontario, contaminating fish resources as far as 250 km downstream [1]. Asubpeeschoseewagong Netum Anishinabek (Grassy Narrows First Nation) and Wabaseemoong Independent Nation (previously known as Whitedog and Islington Bands), for whom walleye *(Sander vitreus)* is central to their traditions, culture, economy and health, were seriously affected by this disaster [2]. Between 1970 and 1997, governmental agencies carried out biomonitoring programs in these communities to assess Hg concentrations in blood and hair [3]. Another program (1970–1992) assessed Hg concentrations in umbilical cord blood collected at the local hospital [3, 4]. Biomarker Hg concentrations from Grassy Narrows First Nation followed a similar pattern to Hg levels in local fish from the contaminated river system, with extremely high concentrations in the early 1970’s, a sharp decline until 1977 and a less pronounced decline until 1987, after which, mean concentrations remained relatively stable [5, 6].

In 1975, neurological examinations of 89 residents from the two communities, carried out by Dr. Masazumi Harada and colleagues [7], showed a high prevalence of signs and symptoms, similar to those reported by patients with Minamata Disease. The team revisited the communities in 2002 and 2004 and examined 175 individuals. Sixty (n=60) persons were diagnosed with Minamata Disease, 54 with Minamata Disease with complications due to other diseases, and 25 with “suspicion of Minamata Disease” [7]. Self-reported symptoms included numbness, pain in limbs, joints and back, decreased vision, impaired hearing, cramps in limbs, dizziness, tendency to fall, forgetfulness, impaired finger movement, tremor and speech impairment. For 27 persons who had been examined in 1975, symptoms had worsened [7]. Takaoka and co-authors [8] compared self-reported symptoms of volunteers from the Grassy Narrows First Nation community from examinations performed in 2010, with residents from the Minamata area and a reference group from other areas of Japan. The authors observed that the prevalence of specific and non-specific complaints of older persons from Grassy Narrows was similar to those from the Minamata area, while the prevalence among younger persons from Grassy Narrows was lower, but higher than the Japanese reference group  [8]. While signs and symptoms reported in this study are consistent with methylmercury poisoning [9], the findings are limited by the recruitment strategy and the absence of measurements of mercury exposure.

In 2015, Grassy Narrows First Nation undertook a Community Health Assessment survey (GN-CHA), which included a list of 59 symptoms, as well as questions on current and past fish consumption. Although understanding one single symptom is worthwhile per se, people rarely present with one symptom, but rather with an array of multiple coexisting symptoms [10]. Clustering bonds concurrently occurring symptoms [10], whose pattern reflects the nature of the underlying dysfunction [11]. Multidimensional approaches and statistical techniques have been proposed to facilitate the integrated analysis of multiple symptoms [10, 12–15]. Also, a growing body of research has used advanced statistical techniques to examine the contributions of Hg exposure [16–26]. The objective of the present study was to bond GN-CHA reported symptoms of nervous system dysfunction into clusters and to examine their association to long-term and past Hg exposure.

## Methods

**Fig. 1:**
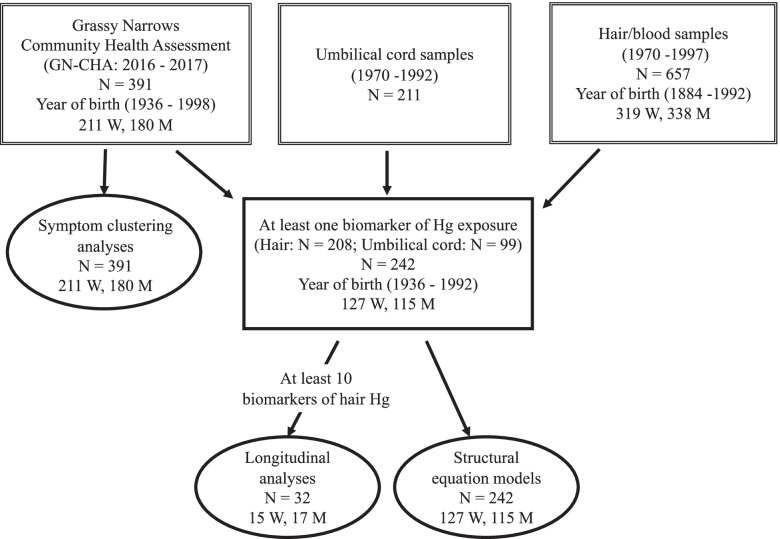
Flow chart of sample size in databases, selection criteria and analyses

For the present study, carried out in collaboration with the Grassy Narrows First Nation community, we merged information from the GN-CHA survey and two government biomarker monitoring programs (umbilical cord Hg and blood and/or hair Hg) to examine the associations between current reported symptoms from the GN-CHA survey and past Hg exposure, using cluster analyses, structural equation models (SEM) and longitudinal mixed effects models (LMEM) (Fig. 1).

### GN-CHA survey

The GN-CHA survey for adults (18 years old and over), initiated in 2015, included 266 questions that covered many aspects of their life and health: demographics, education, generational attendance of residential school, work and income, food consumption, health status, diabetes care, wellness and mental health, injuries, disability, health care access, physical activity, smoking, drinking and drug use, community wellness and traditional culture. Most questions were taken from the First Nation Regional Health Survey 2008/2010 (FNRHS 2008/2010) [27], which provided a basis for comparison with other First Nation communities in Canada. Specific questions were added about fish consumption at different periods of one’s life, as well as illnesses and symptoms consistent with Hg poisoning. The questionnaire was pre-tested with 10 community members in July 2016. A house-to-house survey design was used, with the questionnaire on a web-based platform.

Survey administration was supervised by two field coordinators from Grassy Narrows; nine local interviewers went from house-to-house between December 2016 and March 2017. A total of 213 houses were identified on the reserve; 83.6% of houses were surveyed. Persons living off-reserve were recruited using convenience sampling. The results of the survey were presented to and discussed with community members during several small group and community meetings. The final report was approved by Chief and Council and a summary was made public on May 24, 2018.

A total of 391 Registered Grassy Narrows First Nation members (Band members) participated in the GN-CHA survey. At the time of the survey, 303 were living on-reserve and 88 off-reserve.

For the present study, we used the following variables from the GN-CHA: Demographics: age, sex, living on/off reserve, schooling, generational attendance of residential school in the family. Work: currently working (yes/no), currently looking for work (yes/no), reasons for not working (disability or illness/other). Food security: struggle to pay for food once a month or more in the last 6 months. Fish consumption: childhood fish consumption at 10 years of age (5 categories grouped into once a month or less/at least several times a month); walleye consumption over the past year (not at all/a few times/often). Lifestyle: current smoking (yes/no) and current alcohol consumption (heavy drinking (yes/no), defined in the FNRHS 2008/2010 [27] as 5 drinks in one drinking occasion at least once/month in the past 12 months). Health status: obesity (yes/no) was categorized using Body Mass Index ≥30 kg/m^2^ and at least one reported diagnosed chronic health conditions (yes/no), as listed in the FNRHS 2008/2010 (allergies, arthritis, asthma, cancer, chronic back pain, chronic bronchitis, diabetes, emphysema, heart disease, hepatitis, high blood pressure, liver disease, osteoporosis, rheumatism, stomach or intestinal problems, thyroid problems, and tuberculosis) and at least one diagnosed nervous system disorder (blindness, epilepsy, Bell’s palsy, cerebral palsy, muscular dystrophy, Kennedy’s disease, Parkinson’s diseases, Alzheimer’ disease, senile dementia, psychological/nervous disorders, cognitive/mental disorders, Attention deficit hyperactivity disorders (ADHD), learning disability).

The GN-CHA included 59 self-reported symptoms, rated on a 5-point Likert rating scale (*“Never”*, *“Rarely”*, *“From time to time”*, *“Very often”*, *“All the time”*). Higher scores on the Likert rating scale indicate greater frequency of symptoms.

### Historic Hg biomarker data

At the request of Chief and Council of Grassy Narrows First Nation, the authors obtained from the First Nations and Inuit Health Branch (FNIHB) of the Ministry of Indigenous Services Canada and the Ontario Ministry of Health and Long-term Care, archived Band members’ historic biomarker data (hair and/or blood Hg concentrations), gathered between 1970 and 1997 and umbilical cord Hg data, collected between 1970 and 1992 at the hospital where Grassy Narrows women gave birth (Fig. 1).

Blood and hair samples were taken as part of a monitoring program of the Medical Research Branch of Health Canada [4]. The objective of the program was to identify persons whose Hg biomarker concentrations surpassed the guidelines of the time. There was no sampling strategy. The program primarily targeted fishing guides and their families, but anyone could volunteer and provide a sample [4]. Some years. The sampling period was longer than others and there was no consistency in the month that sampling was carried out.

Samples were analysed for total Hg at Health Canada laboratories [4, 28, 29]. According to Farant et al. 1981 [28], in the initial years, sample analyses were performed according to the Magos method [29] and later, a more efficient and less time-consuming method used an improved cold-vapor atomic absorption technique. The two methods were highly correlated for blood (*r* = 0.98) and hair (*r* = 0.97) [28].

### Past Hg exposure databases

**Fig. 2:**
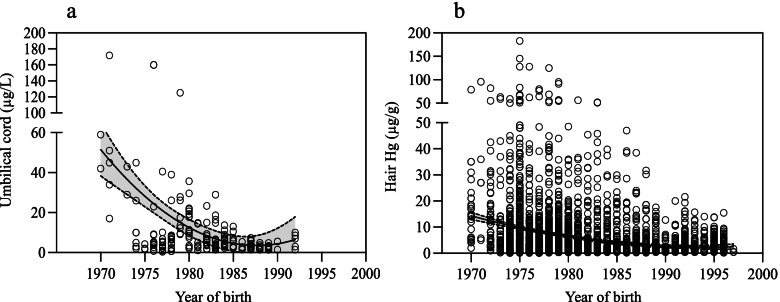
Distribution of Hg biomarker concentrations over time. **a** Umbilical cord blood Hg (μg/L) collected between 1970 and 1992 (*n* = 211). **b** Equivalent hair Hg (μg/g) from samples collected between 1970 and 1997 (*n* = 657)

We created a past Hg exposure database, which included umbilical cord Hg concentrations for 211 newborns from Grassy Narrows, born between 1970 and 1992 (Fig. 2a) and blood/hair monitoring data, collected between 1970 and 1997, from persons born between 1884 and 1992. For the latter, we used the highest measure of equivalent hair Hg concentration for each year sampled; the details are described elsewhere [6]. A total of 3603 year-based equivalent hair measurements were available over the sampling period for 657 persons (Fig. 2b),

For the present study, we selected GN-CHA participants with at least one biomarker measurement: either an umbilical cord Hg measurement (*n* = 99) and/or at least 1 year-based hair Hg concentration (*n* = 208; 1018 measurements) for a total of 242 persons. The number of year-based hair Hg measurements for each person varied between 1 and 23; 75 persons (36.1%) had 5 or more measurements, while 32 (15.4%) had 10 or more. For childhood Hg exposure, we used the mean hair Hg concentration between 5 and 15 years of age (*n* = 137 (56.6%)). Childhood Hg concentrations were significantly higher for persons who reported childhood fish consumption frequency at least several times a month compared to those who ate less (Additional file 1, Fig. 1). Moreover, those who ate fish at least several times a month and had attended a residential school (outside of the community) had lower Hair Hg at 10 years of age (3.8 μg/g [1.7–5.9] (*n* = 16) vs. 5.8 μg/g [4.2–7.3] (*n* = 30)).

### Estimated past Hg exposures

For participants who did not have an umbilical cord Hg measurement, estimated Hg values were predicted from linear regression models using the 211 umbilical cord data (Fig. 2a) on the year of birth for the three periods of Hg exposure (1970–1976, 1977–1987 and 1988–1992). These estimates were then adjusted on where the person’s mother had spent her pregnancy and/or gave birth. For those who were born or whose mother spent her pregnancy in the Wabigoon-English River region between 1970 and 1992, the mean measured umbilical cord blood Hg for their year of birth was used. For those born prior to the Hg discharge in 1962, we attributed 1.0 μg/L for cord blood Hg, while for those born between 1962 and 1970, mean cord blood Hg in 1970 was used (65.7 μg/L), and for those born after 1992, mean cord blood Hg from 1992 was applied (4.48 μg/L). Finally, for individuals, whose mother spent her pregnancy and delivered elsewhere, cord blood Hg was set at 1.0 μg/L.

For study participants, who did not have childhood hair Hg values, we derived estimates from the yearly-hair Hg database (*n* = 657), using the overall mean hair Hg for the year in which they turned 10 years of age. For participants, who were ≥ 10 years old in 1962 (beginning of the discharge) childhood hair Hg was set at 0.1 μg/g. For those who were 10 years of age between 1963 and 1970, the mean measured hair Hg in children in 1970 was used. Since there were few Hg data after 1990 and there appears to be a plateau over this last period (Additional file 1, Fig. 1), we attributed 0.81 μg/g for those who were 10 years of age after 1990. For all those who were 10 years of age after 1962, estimates were then adjusted on reported childhood fish consumption and having attended a residential school. We were unable to estimate childhood hair Hg exposure for 13 persons (5.4%) of the 242 study participants, who were missing hair Hg data between 5 and 15 years old and who did not answer the question about childhood fish consumption in the GN-CHA survey.

To validate the estimated Hg measures, we tested the correlations between measured and estimated values for both umbilical cord and childhood hair Hg, using non-parametric tests (Spearman).

### Symptom clustering

Self-reported symptoms from the GN-CHA survey (*n* = 391) were clustered based on their Likert scale, using a simultaneous two-step multivariate segmentation approach [30–32]. One is agglomerative and aims at maximising a homogeneity criterion by successively aggregating the variables into clusters using a hierarchical ascendant clustering algorithm. The other is representative and creates a composite variable from a weighted linear combination of symptoms (synthetic variable that can be read as a gradient) for each cluster using a mixed factorial approach (PCAMIX method) [30, 32, 33]. For each composite variable (cluster), all participants received a score based on his/her symptom frequency.

A bootstrap approach was used for maximizing the homogeneity criterion within clusters and determining the suitable number of clusters. The desired number of clusters (K) was determined from the analysis of aggregation levels, stability of the partitions via bootstrapped mean-adjusted Rand Index and boxplots. The mean-adjusted Rand index was based on the generation of 60 bootstrap samples. Each cluster was carefully inspected based on its proportion of variation explained (a minimum of 50% was mandatory) and on degree of “closeness” among the symptoms being clustered (a minimum of 0.6 squared correlation of the variable with its composite variable was required). Some symptoms add noise while providing little or no information in identifying the underlying pattern inherent to the cluster [34]. Finally, we tested for the “driving” symptoms that best reflect the cluster. We validated the cluster results, using the VARCLUS function of the SAS computer application (JMP Professional 15.0 software).

A confirmatory factor analysis (CFA) was conducted to evaluate the construct validity of symptom clustering using a series of fit parameters [35–39], using the following indices: Chi-square divided by the degrees of freedom (χ2/df), Standardized Root Mean Square Residual (SRMR), Root Mean Square Error of Approximation (RMSEA), Comparative Fit Index (CFI) and Tucker Lewis Index (TLI). Internal consistency (scale reliability) for each cluster was tested using the Cronbach alpha [35, 40–42]. Construct validity for each cluster was also confirmed by the neurologist (T. S. Lena).

### Structural equation models

SEM is a comprehensive methodology for representing and simultaneously testing a network of complex relationships. This technique can incorporate observed (measured) and unobserved variables (latent), while traditional techniques only allow for measured variables. The latent variable is an unobservable multidimensional construct that cannot be directly measured [43, 44]. SEM allows for data from multiple exposures and multiple outcomes to be considered simultaneously [45]. It is based on an interrelated system of linear regression equations that extend the possibility of relations between observed and latent variables, as well as direct and indirect (mediation) relations and allows for flexibility in modeling covariance structures [19, 39, 46, 47]. Finally, SEM provides path diagrams that are useful to understand and interpret the contribution of the various variables. The standardized factor loadings allow for pathway comparison.

In the SEM, we used the latent variable for symptom clusters rather than the composite variable because it better reflects the structural concept of co-occurring symptoms. A latent variable was likewise used for Hg exposure at different times (prenatal, childhood and having had ≥5 μg/g hair Hg at least once over the monitoring period). Each cluster was analysed using two SEM, the first with the latent Hg exposure variable and the second with the Hg exposure variables at the three different times. We tested possible direct and indirect (mediation) associations between Hg exposure variables and the symptom clusters. Age, sex, year of sampling, residence, as well a series of variables representing socioeconomic status (income, struggle to pay for food), drinking, smoking, obesity and medical history (diabetes, hypertension, heart disease, stroke, hypercholesteremia, separately and/or grouped into the variable “at least one diagnosed chronic health condition”), were tested a priori as covariates. We further tested whether sex modified the relation between past Hg exposures and symptom clusters (moderation). Moderated mediation was also tested with age and sex.

Variables with a skewed distribution were log (base 10) transformed. Because latent variables, Hg exposures and covariates, are all on different scales, we used standardized path coefficients to test the magnitude of relations. Standardized coefficients indicate the expected amount of change in standard deviation (SD) in the dependent latent symptoms cluster for an increase of one SD in the predictor, while other predictors (covariates) are kept constant. The adequacy of model fit to the data was determined using multiple indices: χ2/df, CFI, TLI, RMSEA, SRMR and Akaike’s Information Criterion (AIC) and Schwarz’s Bayesian information criterion (BIC) [35–39]. Diagonally weighted least squares (DWLS) adjustments in SEM were used to take into account the ordinal nature of symptom frequency outcomes. To improve the reliability of the latent construct’s scale or the models, we examined residual variances between indicators. Modification indices (MI) were used to assess the addition or deletion of variables and/or associations to improve the goodness of fit of the SEM. The sequence of initial fit, MI and refitting were repeated until the best fit SEM was attained.

To ensure that the database of 242 participants was sufficient to run SEM, two types of power analyses were performed: i) to detect model misspecification and ii) to detect target effect (influence of Hg exposure on the symptom cluster) [48]. A-priori*,* post-hoc, and compromise power-analyses were run simultaneously to detect model misspecification based on Chi-square likelihood-ratio and RMSEA tests of close and not-close fit, [49–52]. The power for detecting specific target effect was determined with simulated data in SEM [48].

For the SEM, validated prenatal and childhood Hg exposure estimates, described above, were used when there was no measurement, while for GN-CHA variables, missing data were imputed using Multivariate Imputation by Chained Equations (MICE).

To support the SEM, we ran a series of Directed Acyclic Graphs (DAG) for sensitivity analyses. DAG is a visual representation of causal assumptions that makes underlying relations explicit. It is used to detect overt bias, notably through backdoor paths that identify the presence of confounding [53, 54]. Modifications to DAG-implied adjustment sets include conditioning confounding and variables with ambiguous causing roles [55, 56].

### Longitudinal mixed effects models

LMEMs were performed using direct measurements of longitudinal hair Hg with respect to the composite variable for each cluster. We chose mixed effects modeling (MEM) as it is robust to missing data and irregularly spaced sampling; it also handles both time-invariant and time-varying covariates [57]. MEM enables multilevel modeling and partitioning of the covariance structure (random effects). We tested a priori the following covariates (age, sex, variables of lifestyle and medical history, age at sampling and sampling season). The normality of residuals was tested using a q-q plot. The most appropriate model was selected using the Akaike Information Criterion (AIC), the Baysian Information Criteria (BIC) and the likelihood ratio (LR) test at *p* < = 0.05.

For the longitudinal approach, LMEM requires repeated measures. Because of the absence of a biomarker sampling strategy, over half (56%) of the 208 persons with hair Hg measurements had 3 repeated measurements or less. Despite the flexibility of MEM with missing data, we preferred not to impute Hg levels for people whose diet and environmental context varied over time, particularly when living off-reserve. For LMEM, we only included persons with at least 10 hair Hg measurements (*n* = 32 (17 men and 15 women)), with a total of 447 hair Hg measurements, covering a period from 10 to 23 years (median 21 years).

To ensure that there were sufficient observations for the analyses, we estimated the minimal required sample size, based on formulas from Hedeker and co-authors [58] and direct calculations using the G*power software [59–61]. Since one centimeter of a hair sample represents an accumulation of Hg during approximately 1 month [62], we used a low correlation of repeated measures between yearly-based samples (rho 0.1 and 0.2). Because the effect size was unknown, 0.25 was chosen. Power analyses were set at 80%, with a two-tailed 5% hypothesis test, 10 time points and up to four fixed factors; the minimum number of participants required was between 29 and 42.

Threshold of statistical significance in all analyses was set at *p* ≤ 0.05.

Database management and descriptive statistical analyses were performed using JMP Professional 15.0 (Statistical Analysis Hardware (SAS Institute). LMEM were conducted with Stata 16 software (StataCorp. 2019. Stata Statistical Software: Release 16.0. College Station, TX: Stata Corporation). All other analyses were computed using the R statistical software version 3.6.1. (R Core Team, 2016). We used the R package ClustOfVar [31] for clustering analyses and the MICE (Multiple Imputation by Chained Equations) package for multiple imputation of missing data in CFA and SEM. PairedData and dplyr packages were used for paired-t tests. CFA and SEM models were computed with the R package Lavaan [39]. The dagitty and ggdag R packages were used for analysing SEM and DAG. A-priori, post-hoc, and compromise power-analyses were computed using the semPower package. To assess power for detecting a target effect in SEM we used an online tool: yilinandrewang.shinyapps.io/pwrSEM/ [63].

## Results

Relevant characteristics of the 391 participants in the GN-CHA survey, whose data were used for symptom clustering, are presented by age category in Tables 1 and 2. In this community, age also reflects changes in Hg exposure over time. Almost half of the participants were between 30 and 49 years of age, with no difference in age distribution between women and men. There were important age differences in schooling. Proportionally fewer older persons had attended high school, however, over half of those 30 years and older had furthered their education through post-secondary training programs (data not shown). In Canada, First Nation children were placed, often forcibly, in residential schools, with harmful effects on themselves and their families [64, 65]. In the present study, no one born after 1978 had attended residential school; for those born prior to this date, 58 persons (50.0%) had been in a residential school, while for their mothers and/or fathers, it was 95.4%. At the time of the survey, 51.1% of women and 43.1% of men were working, excluding persons studying, retired or at home with children (*n* = 32). Among participants who reported not working, 78% of those between 18 and 49 years of age were looking for work, while 31 persons (68.9%) of those over 30 years of age, who were not looking for work, reported that it was due to illness or disability.

**Table 1:** The relative frequency of the characteristics of the population within each age category

	N^a^	18–29 y n (%)	30–49 y n (%)	50+ y n (%)	Total n (%)	Chi-square (LR)^b^ *p*-value
Sex	391					
Women		66 (53.3%)	97 (51.6%)	48 (60.8%)	211 (54%)	1.94 0.380
Men		58 (46.7%)	72 (48.4%)	31 (39.2%)	180(46%)	
Living on reserve	390	95 (76.6%)	144 (77.0%)	62 (78.5%)	301 (77.2%)	0.10 0.949
Schooling						
Attended school	382	122 (100%)	180 (98.4%)	75 (97.4%)	377 (98.7%)	4.13 0.257
Attended high school	366	115 (98.3%)	166 (94.9%)	48 (64.9%)	329 (89.9%)	52.6 < 0.0001
Other education	386	33 (27.1%)	108 (58.4%)	43 (54.4%)	184 (47.7%)	31.69 < 0.0001
Residential school						
Attended residential school	376	0	6 (3.4%)	52 (66.7%)	58 (15.4%)	171 < 0.0001
Parents in residential school	326	36 (40.5%)	148 (89.7%)	68 (94.4%)	252 (77.3%)	89 < 0.0001
Work activities						
Currently working^c^	349	39 (37.5%)	94 (52.2%)	32 (48.5%)	165 (49.2%)	5.90 0.052
Currently looking for work^c^	165	45 (81.8%)	58 (75.3%)	7 (21.2%)	110 (66.7%)	38.2 < 0.0001
Struggle to pay for food monthly or more in the last year	332	28 (26.9%)	38 (24.2%)	17 (23.9%)	83 (25.0%)	3.65 0.392
Current smoker	376	54 (46.2%)	90 (49.7%)	24 (30.8%)	168 (44.7%)	0.28 0.016
Heavy drinker	360	79 (71.2%)	100 (56.8%)	20 (27.4%)	199 (55.3%)	35.3 < 0.0001
Obese	375	47 (39.8%)	93 (51.7%)	36 (46.8%)	176 (46.9%)	4.03 0.133
At least one diagnosed chronic health condition	385	46 (38.3%)	117 (62.9%)	67 (84.8%)	230 (59.7%)	46.6 < 0.0001
At least one diagnosed nervous system disorder	381	20 (17.1%)	37 (19.9%)	35 (44.9%)	92 (24.2%)	32.6 < 0.0001

^a^Number (N) of valid responses (does not include don’t know or don’t remember and refused)

^b^Likelihood Ratio Chi-square compares the relative frequency between age categories

^c^Excludes students, stay at home parents and retirees

**Table 2:** The relative frequency of fish consumption by age category

	N	18-29 y	30-49 y	50+ y	Total	Chi-square
		n	n	n	n	(LR)
		(%)	(%)	(%)	(%)	*p*-value
Fish consumption at 10 y of age	333					
None		9 (9.4%)	10 (6.1%)	4 (5.5%)	23 (6.9%)	101 <0.0001
Hardly ever/occasionally		32 (33.3%)	26 (15.9%)	3 (4.1%)	61 (18.3%)	
Once a month		24 (25.0%)	38 (23.2%)	2 (2.7%)	64 (19.2%)	
Several times a month		28 (29.2%)	96 (42.1%)	24 (32.9%)	121 (36.4%)	
Every day		3 (3.1%)	21 (12.8%)	40 (54.8%)	64 (19.2%)	
Walleye consumption over past year	382					
Not at all		23 (19.5%)	29 (15.7%)	9 (11.4%)	61 (16.0%)	3.44 0.486
A few times		60 (50.9%)	96 (51.9%)	48 (60.8%)	204 (53.4%)	
Often		35 (29.7%)	60 (32.4%)	22 (27.9%)	117 (30.6%)	

Likelihood Ratio Chi-square compares the relative frequency between age categories

There was no age difference for difficulty paying for food, which was reported by almost 25% of participants. The prevalence of current smokers and heavy drinkers significantly decreased with age. Obesity was most prevalent among those 30–49 years old, of whom two-thirds were women. Sixty percent of participants reported that a health professional had told them that they had at least one chronic health condition; the prevalence increased significantly with age.

There were no differences between men and women for age distribution, schooling, having attended or having parents who attended residential school, difficulty paying for food, and current smoking. Proportionally more men reported heavy drinking compared to women (62.7% vs 48.7%; Chi-square LR 7.17; *p* = 0.007), while women had a significantly higher prevalence of obesity (54.0% vs 38.9%; Chi-square LR: 14.01, *p* = 0.003) and at least one chronic health condition diagnosed by a health professional (68.5% vs 49.7%; Chi-square LR: 14.01, *p* = 0.0001).

In this community, fish, especially walleye, is the source of Hg exposure [4, 5]. Table 2 contains the responses to the GN-CHA questions on past and current fish consumption frequency. Over half of those 50 years and older (born before 1968) reported that, as a child, they ate fish daily. Childhood fish consumption frequency significantly decreased over time. Fish consumption questions were species specific. Walleye is still the fish the most consumed; 30.6% of persons surveyed in the GN-CHA reported often eating walleye compared to 4.7% for whitefish and 3.2% for northern pike. No differences were observed between women and men for the frequency of childhood or current fish consumption.

For symptom clustering of the GN-CHA, aggregation levels and construct stability are presented in Additional file 1, Fig. 2. Aggregation distances supported a cutting threshold between 5 and 8 clusters (Additional file 1, Fig. 2a), while the bootstrapped mean-adjusted R and criterion suggest 6, 7, 11, or 12 (Additional file 1, Fig. 2b). The boxplots (Additional file 1, Fig. 2c) showed that the highest stability of the partition solution varies between K5 and K9, with K7 showing a slightly higher median value, but a lower dispersion around the mean. After verification of all the above criteria, we kept a total of six clusters. Goodness of fit for CFA was acceptable for the six clusters (CFI = 0.85, TLI = 0.84, RMSEA = 0.08, SRMR = 0.05), with a narrow 90% CI, representing a high degree of precision. The Cronbach alpha (> 0.8) for each cluster confirmed internal consistency (Additional file 1, Supplementary Table 1).

The six clusters included a total of 37 symptoms. Additional file 1, Supplementary Table 1 presents the list of symptoms retained in each cluster with its correlation. Cluster 1 (Extrapyramidal impairment), which aggregated the highest number of symptoms (*n* = 9), included tremors, balance impairment, functional limb weakness and pain in arms and legs, reflecting an extension of extrapyramidal neurological symptoms. Cluster 2 (Sensory impairment) included peripheral neuropathic deficits, notably upper and lower limb sensory impairments including numbness, dullness and tingling. Cluster 3 (Cranial nerve disturbances) included anosmia, ageusia, dysphagia and tingling around the mouth. Cluster 4 (Gross motor impairment) aggregated symptoms that affect carrying, lifting and walking. Cluster 5 (Neuro-cognitive deficits) groups cognitive dysfunction associated with memory loss, hearing impairment and speech disorders. Finally, Cluster 6 (Affect/Mood disorders) included anxiety, irritability, depression and sleeping issues. For each cluster, there was no particular driving symptom. Most symptoms were highly related to their own cluster (Additional file 1, Supplementary Table 1).

For the large majority of symptoms, no significant difference was observed in the proportion of men and women who reported its frequency as very often/all the time (Additional file 1, Supplementary Table 1). Symptom frequency increased significantly with age (data not shown), with the exception of difficulty pronouncing words, forgetting to do things, doing nothing, irritability, anxiety, difficulty falling asleep, waking up at night, depressed, tiredness and difficulty concentrating (Fisher’s Exact Test; *p* ≥ 0.08).

The descriptive characteristics of the composite variable for each cluster for women and men are presented in Additional file 1, Table 2. A higher score in the composite variable reflects a higher frequency of coexisting symptoms. Scores were significantly higher in women than in men, with the exception of Neuro-cognitive deficits. Scores increased significantly with age (Rho: 0.138–0.409; *p* <  0.01), with the exception of Affect/Mood disorders (Rho: 0.108; *p* = 0.095).

Of the original GN-CHA participants (*n* = 391) who were used to create the clusters, 242 were selected on the basis of having at least one Hg biomarker measurement (Fig. 1). Umbilical cord, childhood hair Hg concentrations and the percentage of persons with at least one hair mercury concentration ≥ 5 μg/g with respect to age category are presented in Table 3. There was a positive association between childhood fish consumption and measured hair Hg, adjusted for sex and year of sampling (MEM: Wald Chi-square = 96.9; *p* = 0.003). Paired t-tests, comparing mean hair Hg at 10 years of age, for each year, between those who reported eating fish during childhood at least several times a month and those who reported eating less, supported the MEM result (Wilcoxon Signed-Rank prob.>|S|, S: 37.5; *p* = 0.020).

**Table 3:** Measured mercury concentrations by age category

	N	18–29 y Median (min-max)	30–49 y Median (min-max)	50^+^ y Median (min-max)	Total Median (min-max)	Wilcoxon test *p*-value
Umbilical cord (ppb)	99	3.5 (1.5–10)	5.6 (1–78.5)	–	5 (1–78.5)	0.023
Childhood Hair Hg (μg/g)	137	0.9 (0.5–2)	0.7 (0.2–16)	2.9 (0.6–15)	1.1 (0.2–16)	< 0.0001
			n (%)	n (%)	n (%)	
Hair ≥ Hg 5 μg/g at least once	208	0	27 (22.9%)	49 (74.2%)	76 (36.6%)	< 0.0001

Persons retained for the SEM analyses (*n* = 242) had similar characteristics to the original participants (Tables 1 and 2), although they were slightly older (median: 39 years old; interquartile range: 32–50), with proportionally fewer persons in the 18–29 y old category (13.6%). Compared to the entire cohort (Table 2), more young persons reported eating fish frequently during their childhood (41.6%), and often consuming walleye during the past 12 months (41.9%).

In the SEMs, measured and estimated Hg values were used when measures were missing. Validation of estimated Hg values showed significant associations with direct measurements for both prenatal and childhood Hg exposure (*n* = 99; Rho = 0.22; *p* = 0.023 and *n* = 137; Rho = 0.73; *p* < 0.0001, respectively). Most participants (*n* = 197 (81.4%)) were born after 1962 (when exposure began) and had both pre- and post-natal exposures. Only 8 of the 242 persons (3.3%) were over 15 years of age in 1962.

For each symptom cluster, SEMs were performed (i) with latent Hg and (ii) with prenatal, childhood and hair Hg ≥ 5 μg/g. Path diagrams, showing the configuration of the different pathways and the one-to-one relation between variables, are illustrated in Figs. 3 and 4 and in Supplementary Figs. 3–12 in Additional file 1. SEM standardized path coefficients, representing the total quantified contribution of significant direct and indirect associations, are presented in Figs. 5 and 6.

**Fig. 3:**
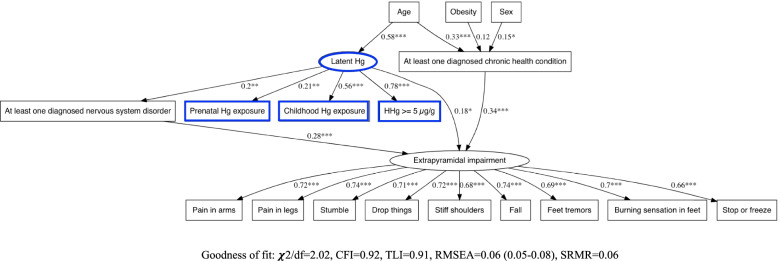
SEM path diagram linking retrospective latent Hg exposure and covariates to the latent symptoms cluster variable for Extrapyramidal impairment. Abbreviations: χ2/df: Chi-square divided by the degrees of freedom (χ2/df), CFI: Comparative Fit Index, TLI: Tucker Lewis Index, RMSEA: Root Mean Square Error of Approximation, SRMR: Standardized Root Mean Square Residual

**Fig. 4:**
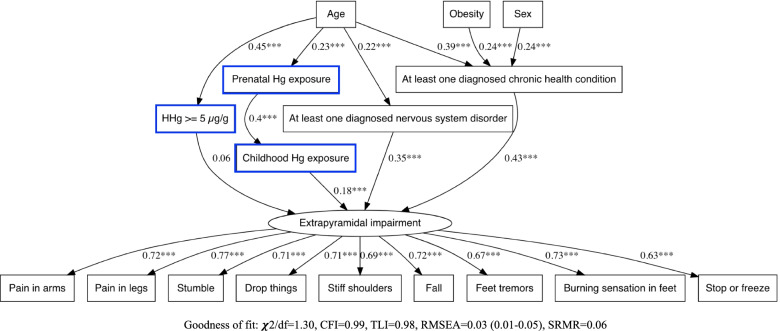
SEM path diagram linking retrospective Hg exposure parameters (prenatal and childhood Hg exposure) and having had ≥5 μg/g hair Hg at least once between 1970 and 1997) and covariates to the latent symptoms cluster variable for Extrapyramidal impairment. Abbreviations: χ2/df: Chi-square divided by the degrees of freedom (χ2/df), CFI: Comparative Fit Index, TLI: Tucker Lewis Index, RMSEA: Root Mean Square Error of Approximation, SRMR: Standardized Root Mean Square Residual

**Fig. 5:**
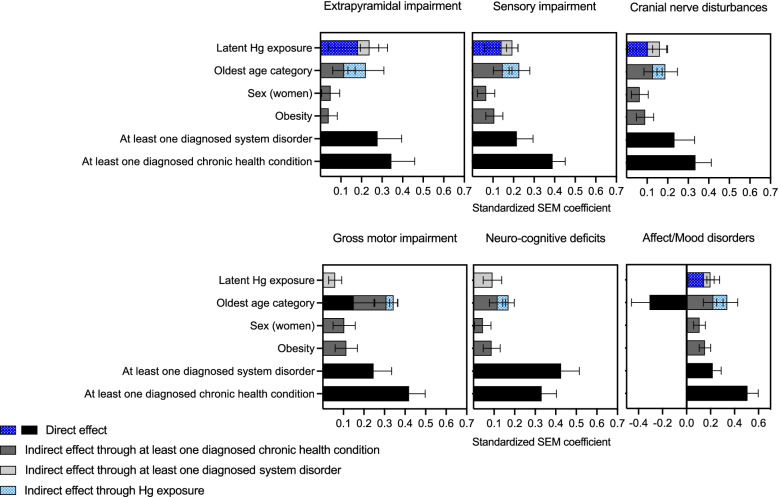
SEM standardized path coefficients for direct and indirect contributions of latent Hg and covariates on the symptom latent variable for each cluster

**Fig. 6:**
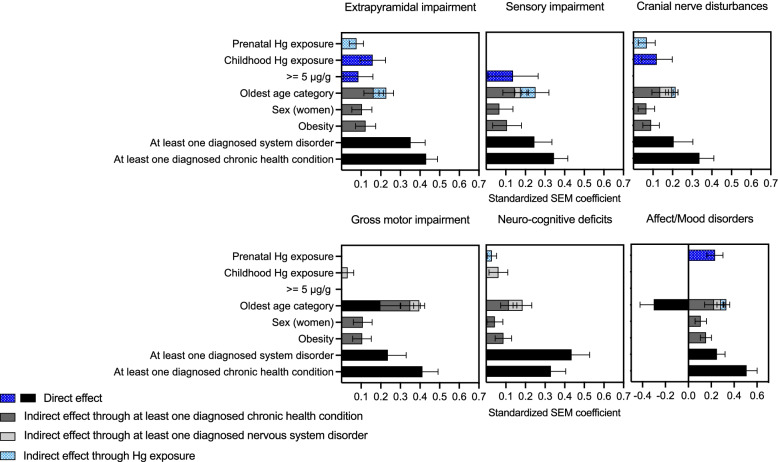
SEM standardized path coefficients for direct and indirect contributions of prenatal, childhood and ≥ 5 μg/g hair Hg (1970–1997) and covariates on the symptom latent variable for each cluster

For all latent Hg-based SEMs, childhood Hg exposure and hair Hg  5 μg/g were the main drivers in the construct of latent Hg (Figs. 3 and 5). Latent Hg showed a direct contribution to most symptom clusters, but not Neuro-cognitive deficits and Gross motor impairment. For all clusters, there was also a pathway mediated through having at least one diagnosed nervous system disorder.

In every SEM with Hg exposure variables for prenatal, childhood and hair Hg ≥ 5 μg/g, there was a significant relation between prenatal and childhood exposure (Figs. 4 and 6 and Supplementary Figs. 8–12, Additional file 1). For Extrapyramidal impairment (Fig. 4) and Cranial nerve disturbances (Supplementary Fig. 9, Additional file 1), standardized path coefficients showed that the contribution of prenatal exposure to the cluster was mediated by childhood exposure.

For Sensory Impairment (Supplementary Fig. 8, Additional file 1), Gross motor impairment (Supplementary Fig. 10, Additional file 1) and Neuro-cognitive deficits (Supplementary Fig. 11, Additional file 1), there was a chain of mediation from prenatal exposure to childhood exposure and from childhood exposure to being diagnosed with at least one nervous system disorder. When the same chain of mediation was modelled for Extrapyramidal impairment and for Cranial nerve disturbances, the models’ fit parameters were weaker. In these cases, the direct association of childhood Hg with the symptom cluster was stronger than its indirect association through diagnosed nervous disorder.

Prenatal Hg exposure was directly associated with Affect/Mood disorders (Supplementary Fig. 12, Additional file 1). Hg ≥ 5 μg/g directly influenced Extrapyramidal and Sensory impairment (Fig. 4 and Supplementary Fig. 8 in Additional file 1, respectively).

The best fit models were those that included childhood exposure as mediator for prenatal exposure. Childhood exposure was not a confounding variable in the relation between prenatal exposure, diagnosed nervous system disorder or any symptom cluster. Moreover, there was no moderated mediation with age category or Hg exposure in the SEM.

Although of different magnitudes, age category, sex and obesity, at least one diagnosed chronic health condition and at least one diagnosed nervous system disorder, were directly and/or indirectly associated with symptom clusters in all SEMs. Positive associations were observed with age category with respect to diagnosed nervous system and chronic health conditions, with the exception of Affect/Mood disorders, for which age was both negatively (direct) and positively (indirect) associated with symptom frequency reporting (Supplementary Fig. 12, Additional file 1). Older persons tended to report fewer symptoms of Affect/Mood disorders (direct), unless they suffered from chronic health conditions and/or nervous system disorders (indirect). Past Hg exposure mediated the contribution of age for all clusters, except for Gross motor impairment (Supplementary Fig. 10, Additional file 1) and Neuro-cognitive deficits (Supplementary Fig. 11, Additional file 1).

Longitudinal MEM analyses were performed with all persons who had at least 10 year-based hair measurements (*n* = 32; 17 men and 15 women). Their median age was 54 years (interquartile range: 48–64). The associations between longitudinal hair Hg and the median of the composite variable for each cluster are shown in Table 4. Age, sex and year of sampling were included as fixed factors, and age at time of sampling as a random factor. In these analyses, 5 of the 6 clusters were significantly associated (*p* ≤ 0.05) or show a tendency (< 0.10) with longitudinal hair Hg. No association was observed between longitudinal hair Hg and Affect/Mood disorders.

**Table 4:** Longitudinal Mixed Effects Model results for estimates of hair Hg concentrations from 1970 to 1997 with respect to the median of the cluster score

Composite cluster variable ^**a, b**^	Hair Hg Estimate^c^	% Confidence Interval	*p* -value
Cluster1 (Extrapyramidal impairment)	1.76	0.0–3.6	0.061
Cluster2 (Sensory impairment)	1.57	−2.7 - 3.4	0.096
Cluster3 (Cranial nerve disturbances)	2.60	1.0–4.2	0.001
Cluster4 (Gross motor impairment)	2.70	0.4–5.1	0.023
Cluster5 (Neuro-cognitive deficits)	2.41	0.6–4.2	0.009
Cluster6 (Affect/Mood disorders)	−0.33	−0.3 - 1.0	0.758

^a^Only participants with 10 measurements and more of hair Hg are included in the analyses (32 persons; 447 Hair Hg measurements)

^b^Significant covariates include age, sex, year of sampling (random effect: age of sampling nested in year of sampling)

^c^Hair Hg estimates represent the difference in hair Hg with respect to cluster scores above and below the median over the sampling period

## Discussion

To our knowledge, this is the first study to link past Hg exposure from freshwater fish consumption with clusters of current multiple coexisting symptoms. Clustering analysis enabled us to characterize the various symptom profiles, which constitute the physical and/or mental translation of structural phenomena. The non-random distribution of symptoms and their strong correlation in each cluster suggests a common mechanism or etiology [10, 12, 66]. Here, the six clusters, derived from statistical empirical validation, reflect the involvement of different aspects of nervous system dysfunction, which we classified as representing primarily Extrapyramidal impairment, Sensory impairment, Cranial nerve disturbances, Gross motor impairment, Neuro-cognitive deficits, and Affect/Mood disorders. The symptoms that make up these clusters were similar to those reported by persons from Grassy Narrows in 2010 [8, 67] and consistent with the many descriptions of methyl Hg poisoning through fish consumption [68–75].

SEM provided the opportunity to simultaneously examine covariance, mediation and moderation between variables through a domino effect of successive pathways from past exposure to current symptoms. The Hg exposure latent variable, which grouped different periods of exposure (prenatal Hg, childhood Hg and hair Hg ≥ 5 μg/g at least once), enabled us to examine their combined contributions. Latent Hg exposure variables have been used in other studies. Choi et al. [23] showed that a grouped Hg exposure variable, including current Hg nail and blood concentrations and hair Hg concentration 7 years previously, was associated with adverse cardiovascular outcomes in Faroese whaling men. In a study by Grandjean and co-authors [76], the combined prenatal and childhood Hg exposures were negatively related to children’s neurobehavioral outcomes. In the present study, the combined past Hg exposures were, directly and indirectly, associated with all clusters.

Hg biomarker data, collected in Grassy Narrows First Nation between 1970 and 1997 provided a unique opportunity to examine symptom clusters with respect to past exposure over a long-term period and at various time points. The findings of longitudinal analyses, based on participants with at least 10 year-based hair Hg measurements over the sampling period, showed that long-term Hg exposure was associated with higher symptom frequency for all the clusters of nervous system dysfunction, with the exception of Affect/Mood disorders. This is consistent with SEM findings where Affect/Mood disorders showed a significant relation only with prenatal exposure.

Independent Hg exposures may provide insight into the relative importance of exposure at different time periods. While prenatal exposure was directly associated with Affect/Mood disorders, for other clusters, its contribution was mediated by childhood exposure. Fetal Minamata patients present with psychiatric disorders (Harada, 1964 cited in Yorifuji et al. [77]). A re-analysis of 1971 data from Minamata patients showed that psychiatric symptoms peaked at 20 years of age [77]. This may be similar to the present study where young adults (≤ 29 years of age) reported a higher frequency of altered mood symptoms. The importance of prenatal and childhood Hg exposure has been raised by many authors [25, 78]. Children are more vulnerable to toxic exposure than adults because their brains are in a state of rapid growth, with relatively higher absorption rates to body weight; their body systems are not prepared to properly metabolize, detoxify, and excrete toxic substances [79–81].

Although the GN-CHA did not include the age of onset of symptoms, the association between past exposure and later-life symptoms raises the issue of delayed and/or progressive neurotoxicity. Delayed neurotoxicity was reported in 13-year-old monkeys, treated with methyl Hg from birth to 7 years of age [82]. A silent latency period for methyl Hg toxicity has been proposed using examples from the Minamata and Iraq disasters [83]. Newland and co-authors [84] discuss mechanisms by which neuronal development may have long-lasting behavioral consequences that appear in adulthood and, in some cases, may not appear until aging.

The FNRHS 2008/2010 included few nervous system disorders in their list of diagnosed health conditions [27]. The GN-CHA added a series of neurologic disorders which share some of the signs and symptoms of Hg poisoning. These disorders mediated the relation between latent Hg exposure and all symptom clusters and between childhood exposure and Sensory impairment, Gross motor impairment and Neuro-cognitive deficits.

In Canada, chronic health conditions are disproportionately higher among Indigenous communities compared to the non-Indigenous population [85]. The most commonly reported chronic health conditions in the GN-CHA are similar to other First Nation communities across Canada: high blood pressure, allergies, arthritis, diabetes, and chronic back pain [27]. Results of the SEM showed the expected associations of having at least one chronic health condition with age and obesity, with women presenting a higher prevalence compared to men. It is noteworthy that the highest direct contribution to all symptom clusters was having at least one diagnosed chronic health condition and/or nervous system disorder.

SEM remains one of the best approaches to assess simultaneously direct and indirect associations by investigating all relevant regression pathways. Our findings are consistent with follow-up studies of persons that had been affected by Hg pollution from the Chisso Company in Minamata, decades after the company had halted the dumping of methyl Hg into the bay [74, 86–88]. The Japanese studies compared persons who had lived in polluted and non-polluted regions since they did not have actual measures of Hg exposure for the individuals who were evaluated.

Some limitations need to be considered in interpreting the present results. The study reposes on self-reported questionnaire data from the GN-CHA, raising the question of recall bias, and under- or over-reporting. To offset these limitations, the Cronbach alpha and CFA confirmed the construct reliability of the clustered symptoms. Reported diagnosed health conditions and nervous system disorders reflected whether a person had consulted a health professional who had diagnosed this condition, as well as the accuracy of the diagnosis. Further studies would benefit from clinical assessments in relation to past Hg exposure.

A further limitation is with respect to Hg biomarkers obtained from a government monitoring program [3]. Sampling was not carried out regularly and samples were not collected yearly from everyone, resulting in many missing values. Moreover, there were seasonal and yearly differences in fish consumption frequency [3, 6]. Hg exposure imprecision could also be associated with blood and hair measurements over the sampling years, notably laboratory measurement imprecision and biological variation [89]. Ideally, one would like to have had continuous Hg biomarker measurements over all participants’ lifetime, but this was not possible. To address the challenges posed by the biomarker database, for the SEMs, the estimated umbilical cord and childhood Hg values were validated, and we used latent variables of Hg exposure to manage residual error terms between variables. For the longitudinal MEMs, we limited the analyses to persons with a minimum of 10 hair Hg measurements, representing exposure for 10 years or more.

Studies on Minamata Disease have shown differences in fetal, non-fetal infantile and adult Hg damage to the brain [90]. In the present study, the large majority of persons experienced both prenatal and postnatal exposure. The mediated contribution of prenatal exposure on symptom clusters through childhood exposure, observed in most SEMs, suggests that their independent contributions are difficult to assess. While both prenatal and childhood Hg exposures decreased over time, reflecting diminishing fish Hg concentration and changing family dietary patterns, they also represent different stages of neurodevelopment and have potentially different effects. Our data showed that childhood Hg exposure may be a mediator/cumulative effect of prenatal exposure, except for Affect/Mood disorders. We tested childhood exposure alone, but the associations were stronger when prenatal exposure was included in the model.

The Grassy Narrows Hg disaster began in 1962 and after 1970, fish Hg concentration and fish consumption decreased over time, paralleled by a decrease in biomarker Hg concentrations. Associations with Hg exposure, observed in the models, may be partially masked by age. The inverse relation between year of birth and Hg exposure made age an ambiguous variable. Age was a risk factor for the symptom clusters, covaried with Hg exposure, but was not a consequence of Hg exposure.

## Conclusions

Since the disaster, Grassy Narrows First Nation has expressed concern about the high rate of symptoms of nervous system dysfunction in their community. This is the first study to demonstrate links between long-term and past Hg exposure and current coexisting symptoms. Given the complexity of the interrelations between the various determinants of chronic health problems in this and other First Nation communities, SEM and LMEM methods were useful to link past Hg exposure with present-day symptoms. Further studies should examine the mechanisms that underlie these manifestations.

## Supplementary Information

**Additional file 1: Supplementary Table 1.** Characteristics of symptom clusters.

## Data Availability

Archived mercury biomarker data were obtained from the First Nations and Inuit Health Branch (FNIHB) of the Ministry of Indigenous Services Canada, at the request of Grassy Narrows First Nation. The datasets generated and analysed in the present study are the property of Grassy Narrows First Nation. Permission for use of the data lies with Grassy Narrows Chief and Council.

## Abbreviations

Hg: mercury
GN-CHA: Grassy Narrows Community Health Assessment
FNRHS: First Nations Regional Health Survey
FNIHB: First Nations and Inuit Health Branch
SEM: Structural Equation Model
LMEM: Longitudinal Mixed Effects Model
CFA: Confirmatory factor analysis (CFA)
χ2/df: Chi-square divided by the degrees of freedom
SRMR: Standardized Root Mean Square Residual
RMSEA: Root Mean Square Error of Approximation
CFI: Comparative Fit Index
TLI: Tucker Lewis Index
AIC: Akaike’s Information Criterion
BIC: Schwarz Bayesian Information Criterion
DWLS: Diagonally weighted least squares
MI: Modification Indices
MICE: Multivariate Imputation by Chained Equations
DAG: Directed Acyclic Graphs
MEM: Mixed Effects Model
