# Supplementary material for: Past mercury exposure and current symptoms of nervous system dysfunction in adults of a First Nation community (Canada)
Source: Environ Health. 2022 Mar 16;21:34. doi: 10.1186/s12940-022-00838-y (PMC8925187; doi:10.1186/s12940-022-00838-y)
Supplement: Supplementary file 1 — Additional file 1: Supplementary Table 1. Characteristics of symptom clusters. [file 12940_2022_838_MOESM1_ESM.pdf]

Additional File 1

Supplementary Table 1. Characteristics of symptom clusters

	Chronbach alpha (IC 95%)	Correlation within cluster	Reported Very often or All the time			Difference Chi-square/ Fisher's Exact test p-value
			Overall	Women	Men	
**Cluster 1: Extrapyramidal Impairment**						
	0.91 (0.90-0.92)					
Pain in arms		0.80	44 (11.5%)	28 (13.7%)	16 (9.0%)	0.19
Pain in legs		0.80	75 (19.6%)	52 (25.2%)	23 (13.1%)	0.003
Drop things		0.75	53 (13.9%)	37 (17.9%)	16 (9.2%)	0.017
Feet tremors		0.77	25 (6.6%)	13 (6.4%)	12 (6.8%)	0.873
Stiff shoulders		0.75	51 (13.4%)	33 (16.1%)	18 (10.2%)	0.099
Burning feet		0.75	48 (12.7%)	34 (16.8%)	14 (8.0%)	0.01
Stumble		0.78	39 (10.1%)	21 (10.2%)	18 (10.1%)	0.992
Stop or freeze		0.74	14 (3.7%)	10 (4.9%)	4 (2.3%)	0.275
Fall		0.75	19 (5.0%)	14 (6.8%)	5 (2.8%)	0.099

	Chronbach alpha (IC 95%)	Correlation within cluster	Reported Very often or All the time			Difference Chi-square/ Fisher's Exact test p-value
			Overall	Women	Men	
**Cluster 2: Sensory Impairment**						
	0.94 (0.93-0.95)					
Numbness in hands		0.88	53 (14.0%)	37 (18.1%)	16 (9.1%)	0.01
Numbness in feet		0.89	56 (14.7%)	34 (16.5%)	22 (12.5%)	0.268
Dull feeling in hands		0.88	44 (11.7%)	27 (13.3%)	17 (9.8%)	0.285
Dull feeling in feet		0.87	46 (12.1%)	30 (14.6%)	16 (9.1%)	0.102
Tingling in hands		0.87	54 (14.2%)	33 (16.1%)	21 (12.0%)	0.243
Tingling in feet		0.87	53 (13.8%)	34 (16.5%)	19 (10.7%)	0.1

	Chronbach alpha (IC 95%)	Correlation within cluster	Reported Very often or All the time			Difference Chi-square/ Fisher's Exact test p-value
			Overall	Women	Men	
**Cluster 3 : Cranial nerve disturbances**						
	0.85 (0.83-0.88)					
Loss of taste		0.85	23 (6.0%)	17 (8.3%)	6 (3.4%)	0.053
Loss of smell		0.82	26 (6.8%)	16 (7.8%)	10 (5.7%)	0.423
Difficulty swallowing		0.81	33 (8.6%)	23 (11.2%)	10 (5.7%)	0.068
Tingling around mouth		0.73	24 (6.3%)	17 (8.3%)	7 (4.0%)	0.094
Choking		0.81	15 (3.9%)	12 (5.8%)	3 (1.7%)	0.061

	Chronbach alpha (IC 95%)	Correlation within cluster	Reported Very often or All the time			Difference Chi-square/ Fisher's Exact test p-value
			Overall	Women	Men	
**Cluster 4: Gross motor impairment**						
	0.89 (0.88-0.91)					
Difficulty walking 5 minutes without rest		0.91	38 (9.9%)	29 (14.0%)	9 (5.1%)	0.004
Difficulty climbing stairs		0.92	47 (12.3%)	37 (17.8%)	10 (5.68%)	0
Trouble lifting		0.89	77 (20.3%)	50 (24.5%)	27 (15.3%)	0.025
Trouble lifting 10 lbs		0.82	36 (9.5%)	32 (15.5%)	4 (2.3%)	0

	Chronbach alpha (IC 95%)	Correlation within cluster	Reported Very often or All the time			Difference Chi-square/ Fisher's Exact test p-value
			Overall	Women	Men	
**Cluster 5: Neuro-cognitive deficits**						
	0.82 (0.79-0.85)					
Trouble hearing		0.74	48 (12.5%)	28 (13.6%)	20 (11.2%)	0.485
Difficulty having his/her speech understood		0.77	27 (7.1%)	17 (8.3%)	10 (5.7%)	0.424
Difficulty pronouncing words		0.77	39 (10.1%)	21 (10.1%)	18 (10.1%)	0.992
Forget where to put things		0.79	76 (19.8%)	50 (24.3%)	26 (14.7%)	0.018
Forget doing things		0.8	35 (9.1%)	23 (11.1%)	12 (6.8%)	0.158

	Chronbach alpha (IC 95%)	Correlation within cluster	Reported Very often or All the time			Difference Chi-square/ Fisher's Exact test p-value
			Overall	Women	Men	
**Cluster 6: Affect/Mood disorders**						
	0.9 (0.89-0.91)					
Anxious		0.81	57 (14.9%)	39 (18.9%)	18 (10.2%)	0.016
Tired		0.81	111 (28.9%)	74 (35.9%)	37 (20.9%)	0.001
Irritable		0.81	50 (13.1%)	36 (17.5%)	14 (7.9%)	0.006
Depressed		0.77	58 (15.4%)	36 (17.9%)	22 (12.6%)	0.151
Difficulty concentrating		0.78	60 (15.9%)	39 (19.2%)	21 (12.0%)	0.054
Wake up at night		0.77	141 (36.8%)	86 (41.6%)	55 (31.2%)	0.037
Trouble falling asleep		0.75	138 (36.0%)	74 (35.8%)	64 (36.4%)	0.901
Doing nothing		0.72	65 (17.0%)	45 (21.9%)	20 (11.2%)	0.005

Supplementary Table 2. Descriptive characteristics of the composite variable in each cluster

Clusters	Component Score Mean, Median (min-max)			Wilcoxon p-value
	All	Women	Men	
Cluster 1 (Extrapyramidal impairment)	2.7, 2.2 (0-11.2)	3.0, 2.6 (0-1.2)	2.3, 1.7 (0-9.1)	0.002
Cluster 2 (Sensory impairment)	2.6, 2.4 (0-8.3)	2.8, 2.8 (0-8.3)	2.3, 2.1 (0-8.3)	0.012
Cluster 3 (Cranial nerve disturbances)	1.4, 0.9 (0-7.9)	1.6, 1.0 (0-7.9)	1.1, 0.4 (0-6.9)	0.017
Cluster 4 (Gross motor impairment)	1.4, 0.7 (0-6.6)	1.8, 1.1 (0-6.6)	1.0, 0.3 (0-6.2)	0.000
Cluster 5 (Neuro-cognitive deficits)	2.3, 2.1 (0-8.0)	2.4, 2.3 (0-8.0)	2.1, 2.0 (0-7.2)	0.199
Cluster 6 (Affect/Mood disorders)	4.3, 4.3 (0-10.4)	4.7, 4.9 (0-10.4)	3.8, 4.0 (0-10.1)	0.000

Supplementary Figure 1. Equivalent mean childhood hair Hg between 5 and 15 years of age with respect to reported childhood fish consumption at around 10 years old (n = 123).

Note: The fitted line represents second order polynomial (quadratic), with a shaded confidence band.

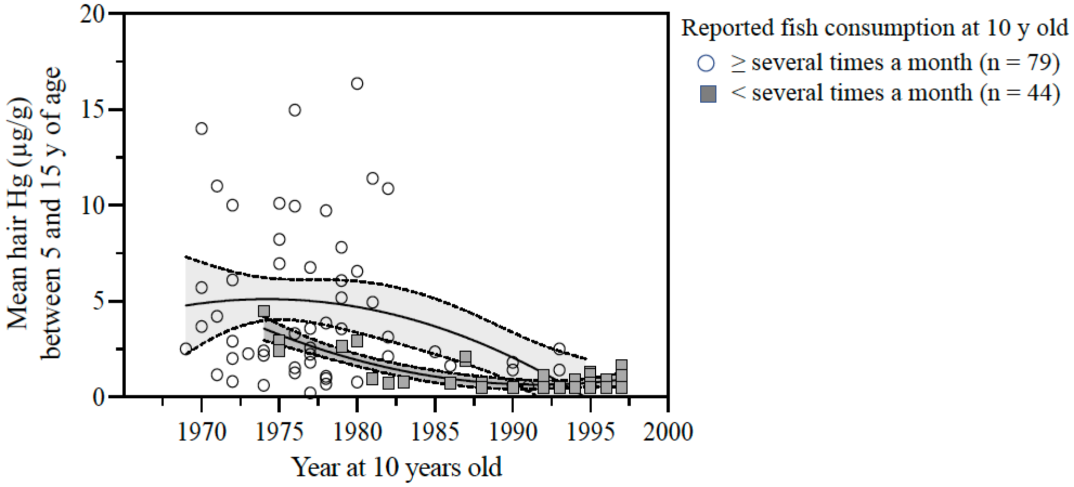

Supplementary Figure 2. Graphic outputs for cluster decision

2a. Plot of the aggregation levels of symptom clusters

2b. Plot of stability of the partitions of clusters

2c. Plot of dispersion of the adjusted Rand index

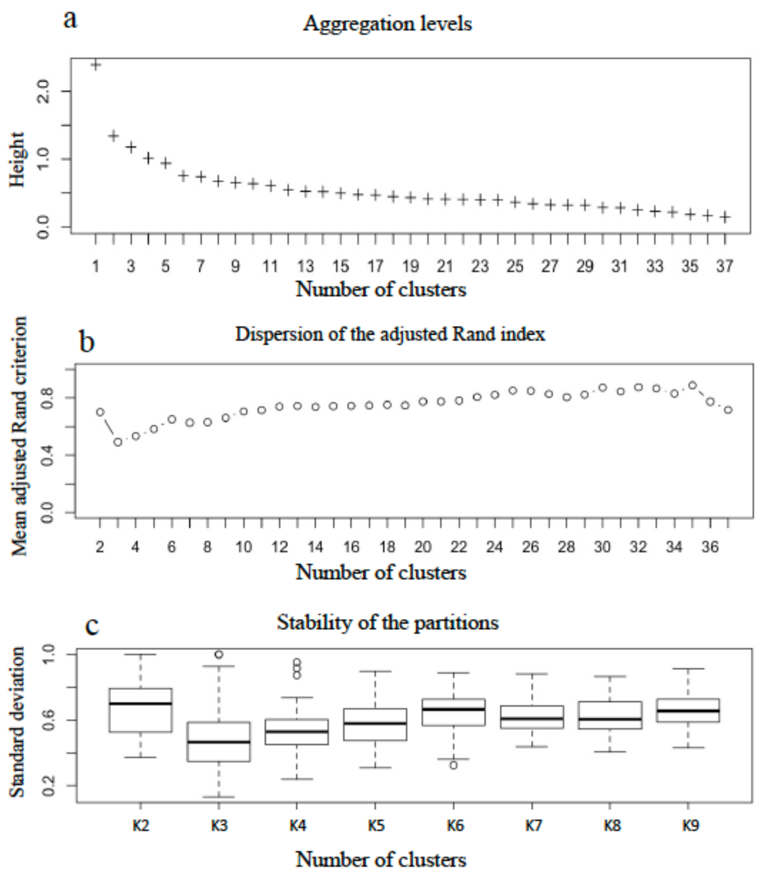

Supplementary Figures 3-7. SEM path diagrams linking retrospective latent Hg exposure and covariates to the latent symptoms cluster variables for Sensory Impairment (3), Cranial nerve disturbances (4), Gross motor impairment (5), Neuro-cognitive deficits (6) and Affect/Mood disorders (7). The latent variable (Latent Hg) binds prenatal, childhood Hg exposure and having had ≥ 5µg/g hair Hg at least once between 1970 and 1997.

Abbreviations: $\chi 2$/df: Chi-square divided by the degrees of freedom ($\chi 2$/df), CFI: Comparative Fit Index, TLI: Tucker Lewis Index, RMSEA: Root Mean Square Error of Approximation, SRMR: Standardized Root Mean Square Residual.

Supplementary Figures 8-12. SEM path diagrams linking retrospective Hg exposure parameters (prenatal, childhood Hg exposure, and having had 5µg/g hair Hg exposure at least once between 1970 and 1997) and covariates with latent symptoms cluster variables for Sensory Impairment (8), Cranial nerve disturbances (9), Gross motor impairment (10), Neuro-cognitive deficits (11), Affect/Mood disorders (12).

Abbreviations: $\chi 2$/df: Chi-square divided by the degrees of freedom ($\chi 2$/df), CFI: Comparative Fit Index, TLI: Tucker Lewis Index, RMSEA: Root Mean Square Error of Approximation, SRMR: Standardized Root Mean Square Residual.

Supplementary Figure 3. SEM path diagram linking retrospective latent Hg exposure and covariates to the latent symptoms cluster variable for Sensory impairment

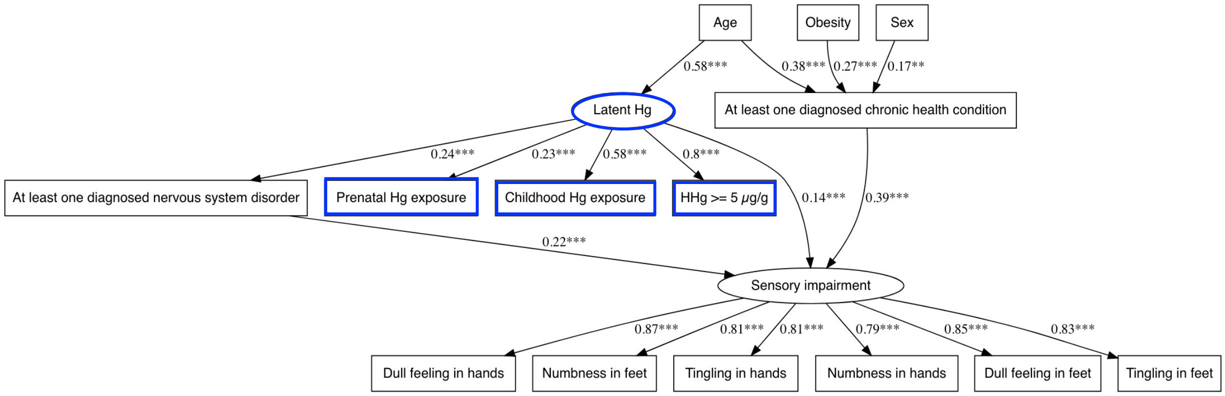

Goodness of fit: χ2/df=1.00, CFI=1.00, TLI=1.00, RMSEA=0.0 (0.01-0.04), SRMR=0.05

Supplementary Figure 4. SEM path diagram linking retrospective latent Hg exposure and covariates to the latent symptoms cluster variable for Cranial nerve disturbances

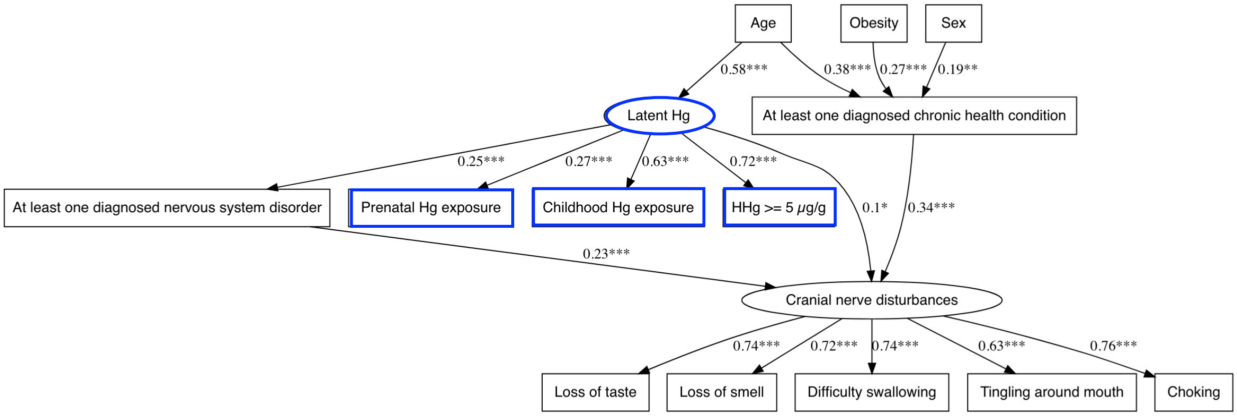

Goodness of fit: $\chi 2$/df=1.27, CFI=0.98, TLI=0.97, RMSEA=0.03 (0.00-0.05), SRMR=0.06

Supplementary Figure 5. SEM path diagram linking retrospective latent Hg exposure and covariates to the latent symptoms cluster variable for Gross motor impairment

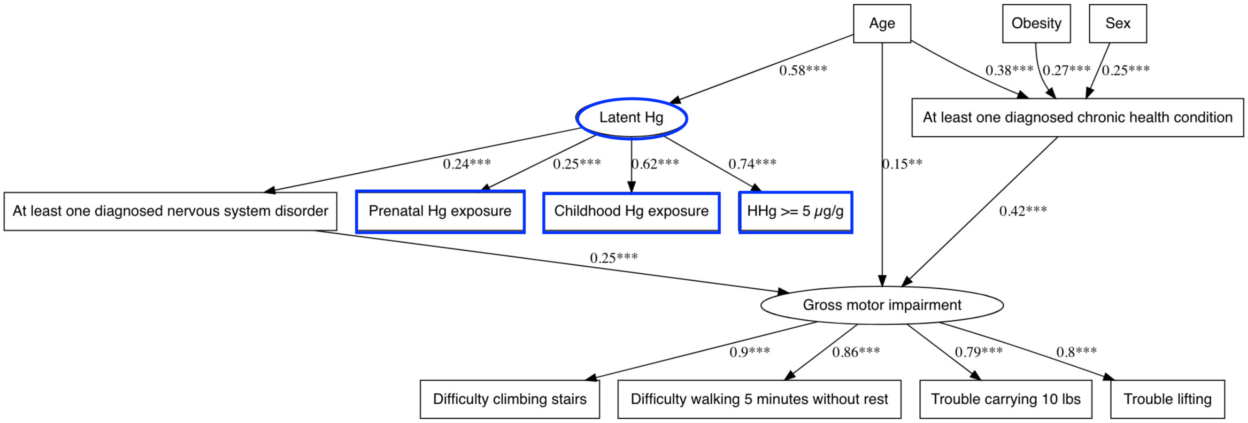

Goodness of fit: $\chi 2$/df=1.46, CFI=0.98, TLI=0.97, RMSEA=0.04 (0.02-0.06), SRMR=0.06

Supplementary Figure 6. SEM path diagram linking retrospective latent Hg exposure and covariates to the latent symptoms cluster variable for Neuro-cognitive deficits

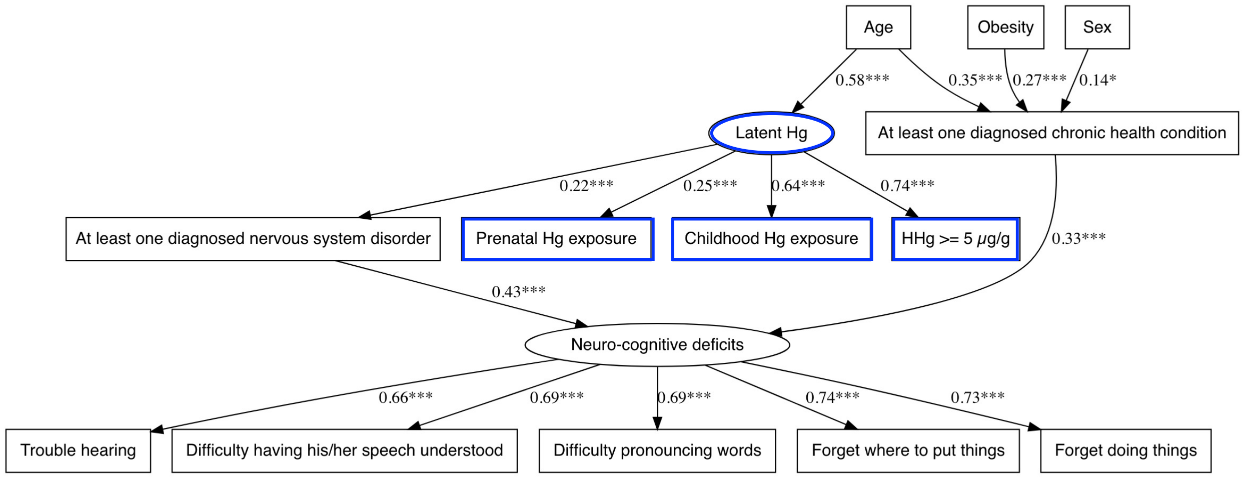

Goodness of fit: $\chi 2$/df=1.45, CFI=0.97, TLI=0.96, RMSEA=0.04 (0.02-0.06), SRMR=0.06

Supplementary Figure 7. SEM path diagram linking retrospective latent Hg exposure and covariates to the latent symptoms cluster variable for Affect/Mood disorders

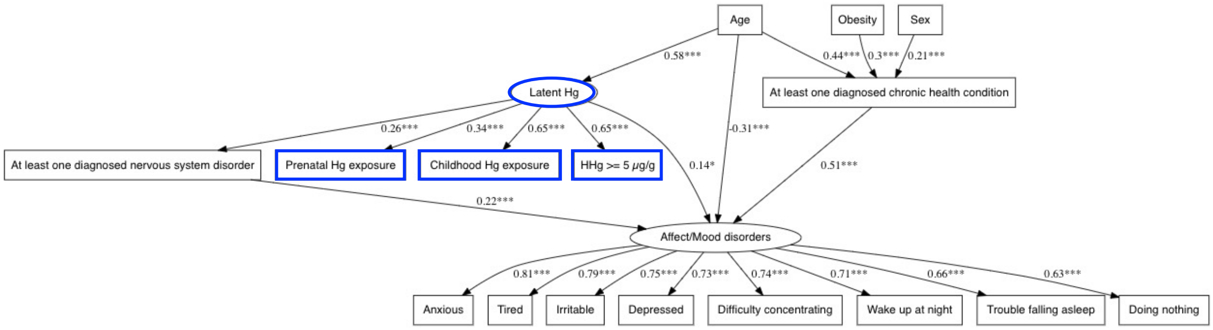

Goodness of fit: $\chi 2$/df=1.33, CFI=0.99, TLI=0.98, RMSEA=0.04 (0.02-0.05), SRMR=0.06

Supplementary Figure 8. SEM path diagram linking retrospective Hg exposure parameters (prenatal and childhood Hg exposure, and having had ≥ 5µg/g hair Hg at least once between 1970 and 1997) and covariates to the latent symptoms cluster variable for Sensory impairment

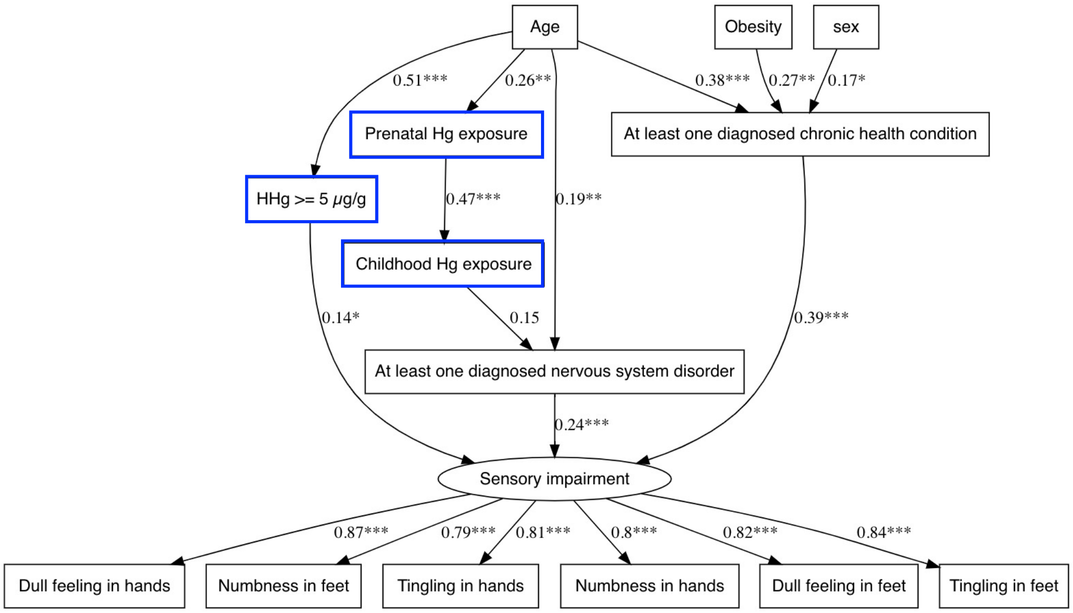

Goodness of fit: χ2/df=1.72, CFI=0.97, TLI=0.97, RMSEA=0.05 (0.04-0.07), SRMR=0.07

Supplementary Figure 9. SEM path diagram linking retrospective Hg exposure parameters (prenatal and childhood Hg exposure, and having had ≥ 5µg/g hair Hg at least once between 1970 and 1997) and covariates to the latent symptoms cluster variable for Cranial nerve impairment

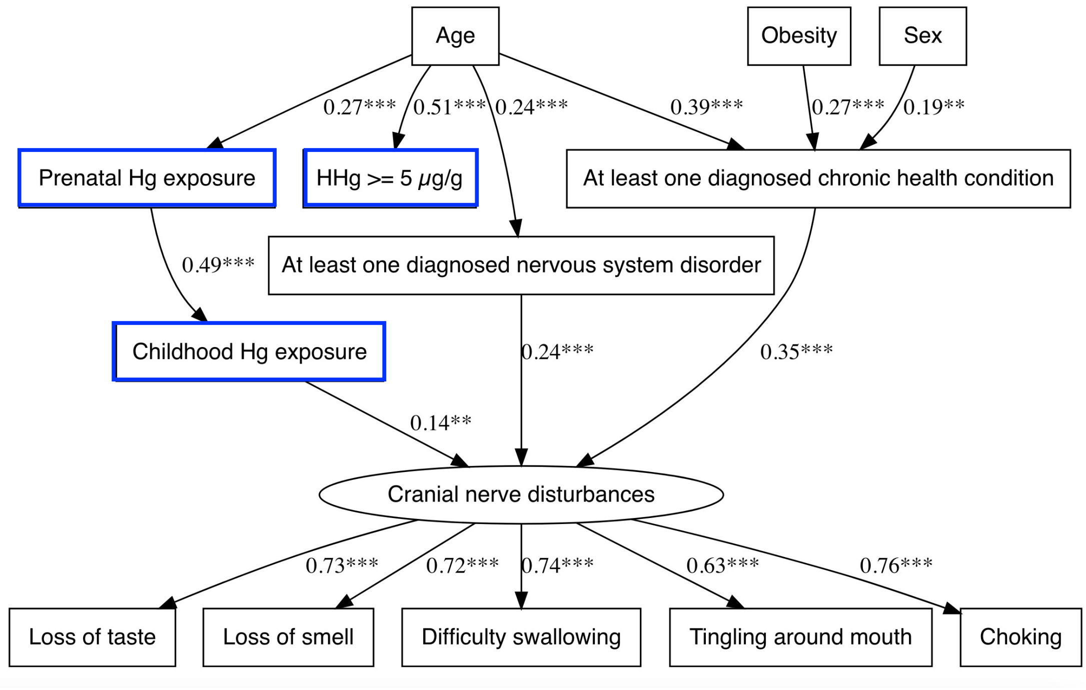

Goodness of fit: $\chi 2$/df=2.05, CFI=0.92, TLI=0.90, RMSEA=0.07 (0.05-0.08), SRMR=0.07

Supplementary Figure 10. SEM path diagram linking retrospective Hg exposure parameters (prenatal and childhood Hg exposure, and having had ≥ 5µg/g hair Hg at least once between 1970 and 1997) and covariates to the latent symptoms cluster variable for Gross motor impairment

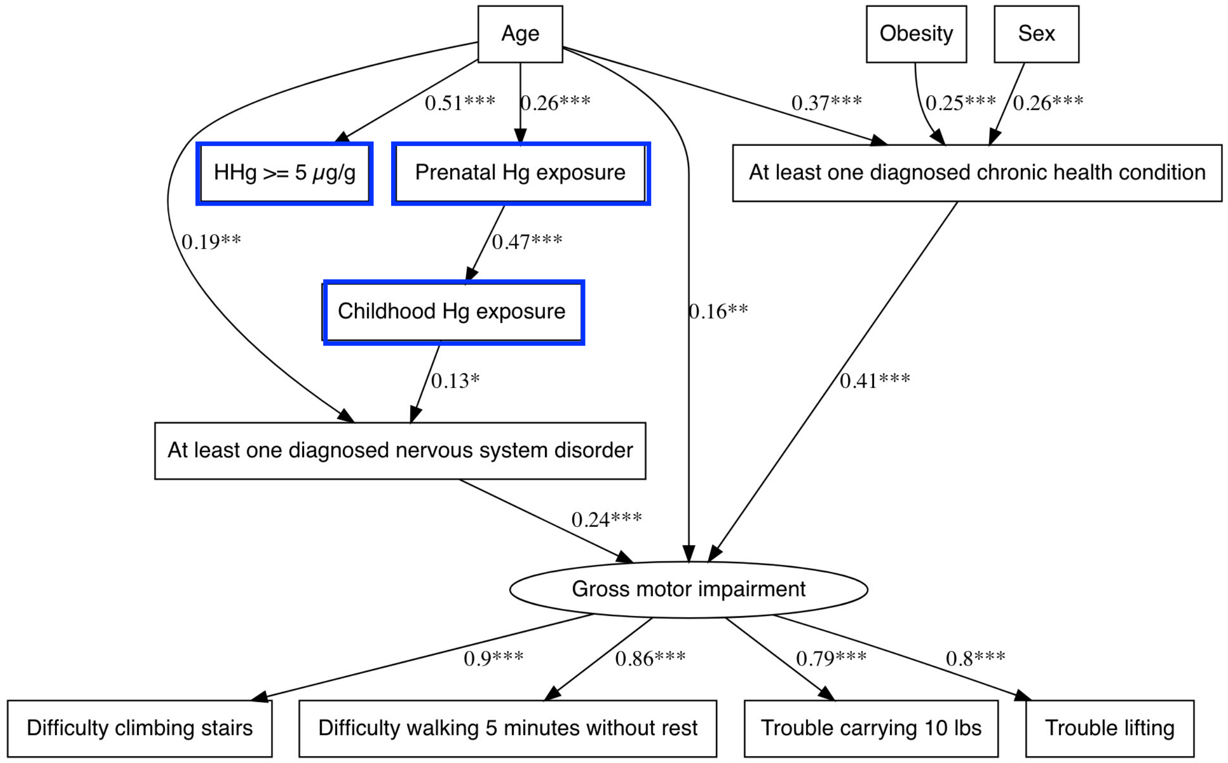

Goodness of fit: $\chi 2$/df=2.47, CFI=0.93, TLI=0.91, RMSEA=0.08 (0.06-0.09), SRMR=0.08

Supplementary Figure 11. SEM path diagram linking retrospective Hg exposure parameters (prenatal and childhood Hg exposure, and having had ≥ 5µg/g hair Hg at least once between 1970 and 1997) and covariates to the latent symptoms cluster variable for Neuro-cognitive deficits

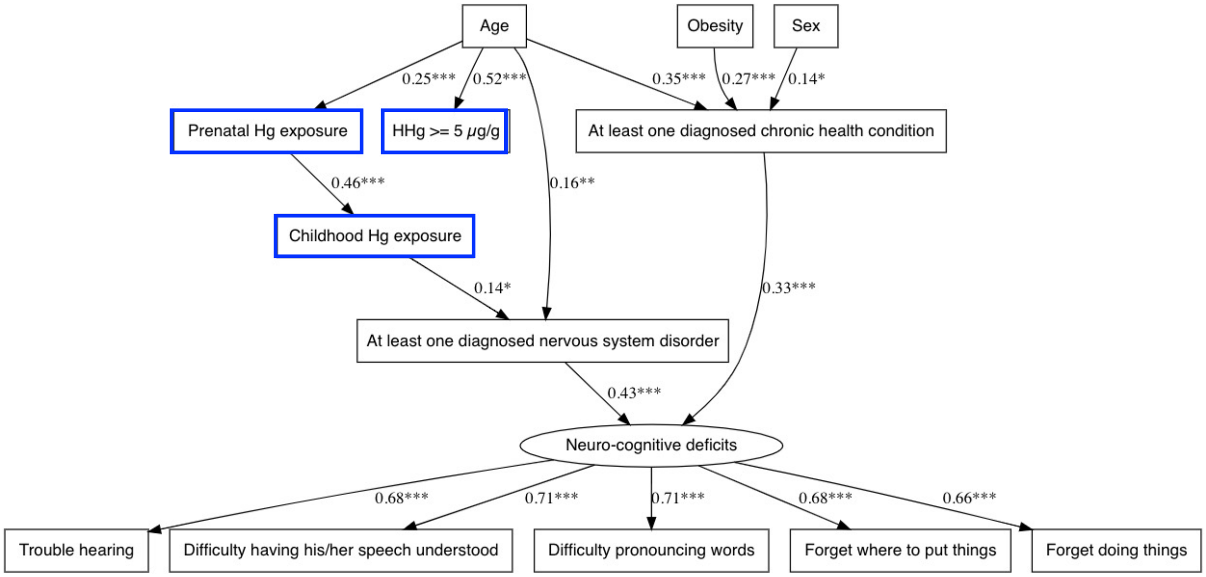

Goodness of fit: $\chi 2$/df=2.23, CFI=0.92, TLI=0.90, RMSEA=0.07 (0.05-0.09), SRMR=0.08

Supplementary Figure 12. SEM path diagram linking retrospective Hg exposure parameters (prenatal and childhood Hg exposure, and having had ≥ 5μg/g hair Hg at least once between 1970 and 1997) and covariates to the latent symptoms cluster variable for Affect/Mood disorders

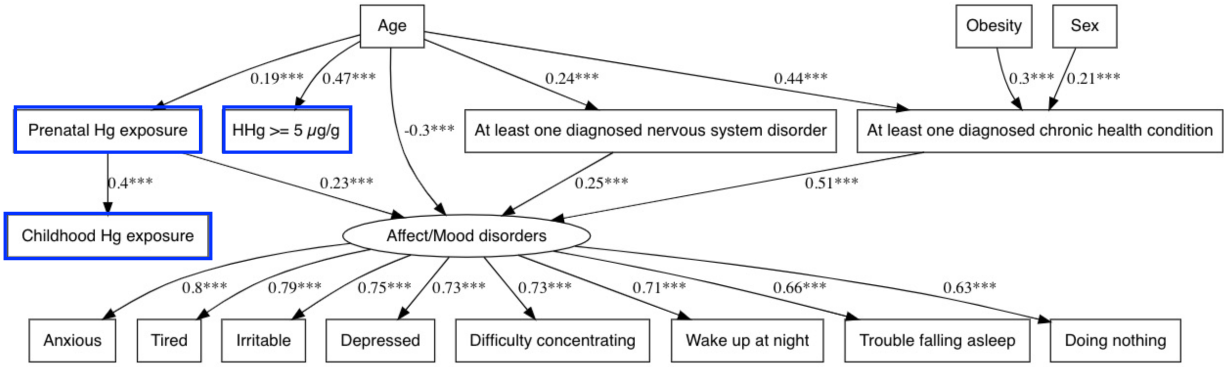

Goodness of fit: $\chi 2$/df=1.07, CFI=0.99, TLI=0.98, RMSEA=0.02 (0.00-0.04), SRMR=0.05
